# Monolayer graphene/GaN heterostructure photodetector with UV-IR dual-wavelength photoresponses

**DOI:** 10.1007/s12200-024-00121-7

**Published:** 2024-06-07

**Authors:** Junjun Xue, Jiaming Tong, Zhujun Gao, Zhouyu Chen, Haoyu Fang, Saisai Wang, Ting Zhi, Jin Wang

**Affiliations:** 1 College of Electronic and Optical Engineering & College of Flexible Electronics (Future Technology), Nanjing University of Posts and Telecommunications, Nanjing, 210023 China; 2 Portland Institute, Nanjing University of Posts and Telecommunications, Nanjing, 210023 China

**Keywords:** Wide bandgap semiconductors, Graphene, Dual-wavelength, Photodetector

## Abstract

An ultraviolet-infrared (UV-IR) dual-wavelength photodetector (PD) based on a monolayer (ML) graphene/GaN heterostructure has been successfully fabricated in this work. The ML graphene was synthesized by chemical vapor deposition (CVD) and subsequently transferred onto GaN substrate using polymethylmethacrylate (PMMA). The morphological and optical properties of the as-prepared graphene and GaN were presented. The fabricated PD based on the graphene/GaN heterostructure exhibited excellent rectify behavior by measuring the current–voltage (*I–V*) characteristics under dark conditions, and the spectral response demonstrated that the device revealed an UV-IR dual-wavelength photoresponse. In addition, the energy band structure and absorption properties of the ML graphene/GaN heterostructure were theoretically investigated based on density functional theory (DFT) to explore the underlying physical mechanism of the two-dimensional (2D)/three-dimensional (3D) hybrid heterostructure PD device. This work paves the way for the development of innovative GaN-based dual-wavelength optoelectronic devices, offering a potential strategy for future applications in the field of advanced photodetection technology.

**Graphical Abstract:**

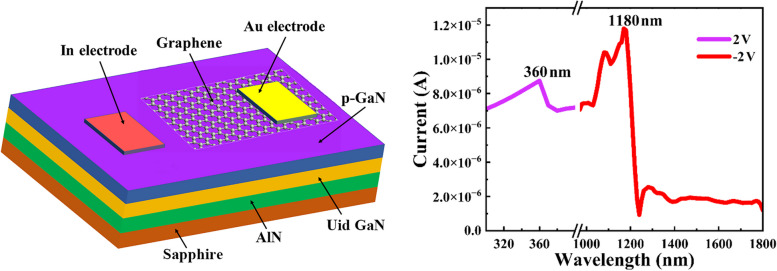

## Introduction

With the development of wide band gap semiconductor epitaxial technology and energy band theories, ultraviolet-infrared (UV-IR) dual-wavelength GaN-based photodetectors (PDs) have attracted great attention because of their characteristics of reducing false alarm rate and suppressing background noise [1–5]. The original UV-IR dual-wavelength PD was packaged by two independent UV and IR detectors, which is not only large in size, but also high in cost [6]. Therefore, in order to realize the real UV-IR monolithic integration, a variety of GaN-based UV-IR two-color PDs are proposed. Singh et al. [7] proposed a broadband PD based on MoS_2_/GaN/Si heterojunction, which realized wavelength selection in UV and near-infrared (NIR) regions by photocurrent polarity reversal. Sandhu et al. [8] proposed a MoSe_2_/GaN heterojunction PD to obtain the response from UV to IR. An UV-NIR dual-wavelength PDs fabricated by using p-GaN/α-In_2_Se_3_ vertical heterostructures were demonstrated and exhibited excellent transient photoresponses [9]. However, the unsatisfactory IR properties and expensive preparation process limit the development of GaN-based UV-IR dual-wavelength PDs [1, 10].

GaN has been widely used in PD and semiconductor power devices because of its high electron saturation drift velocity, wide band gap, as well as excellent chemical and thermal stability [11–14]. Nevertheless, the low conductivity and large defect density of GaN limit the photocarrier collection efficiency of traditional GaN-based PDs [15]. Graphene/GaN heterostructure can exactly overcome these problems [15, 16]. Two-dimensional (2D) graphene has unique properties such as optical transmittance of about 97.7%, broad spectral absorption, high carrier mobility, short carrier lifetime, and extraordinary mechanical flexibility [17–21]. The proposal of 2D graphene/semiconductor heterostructure opens a new way for the development of PD technologies [22].

Therefore, in this work, we propose a 2D monolayer (ML) graphene/three-dimensional (3D) p-GaN heterostructure to construct the PD devices. The heterostructure fully utilizes the excellent optoelectronic properties of 2D graphene and bulk GaN. To better understand the unique optoelectronic properties of graphene/p-GaN heterostructure, we studied current–voltage (*I–V*) characteristics and spectral response of the constructed PD. Meanwhile, the energy band structure and optical properties of the ML graphene/GaN heterostructure were theoretically investigated by density functional theory (DFT). Moreover, 2D graphene/3D p-GaN hybrid heterostructure device exhibits dual-wavelength photoresponse in UV-IR regions, which is expected to be an important candidate for the realization of GaN-based dual-wavelength PDs. Generally, the dual-wavelength detection not only can reduce the detection errors in light communication field, but also provides more solutions for imaging, sensing, flame monitoring and other fields in the future [23, 24].

## Experimental details

**Fig. 1:**
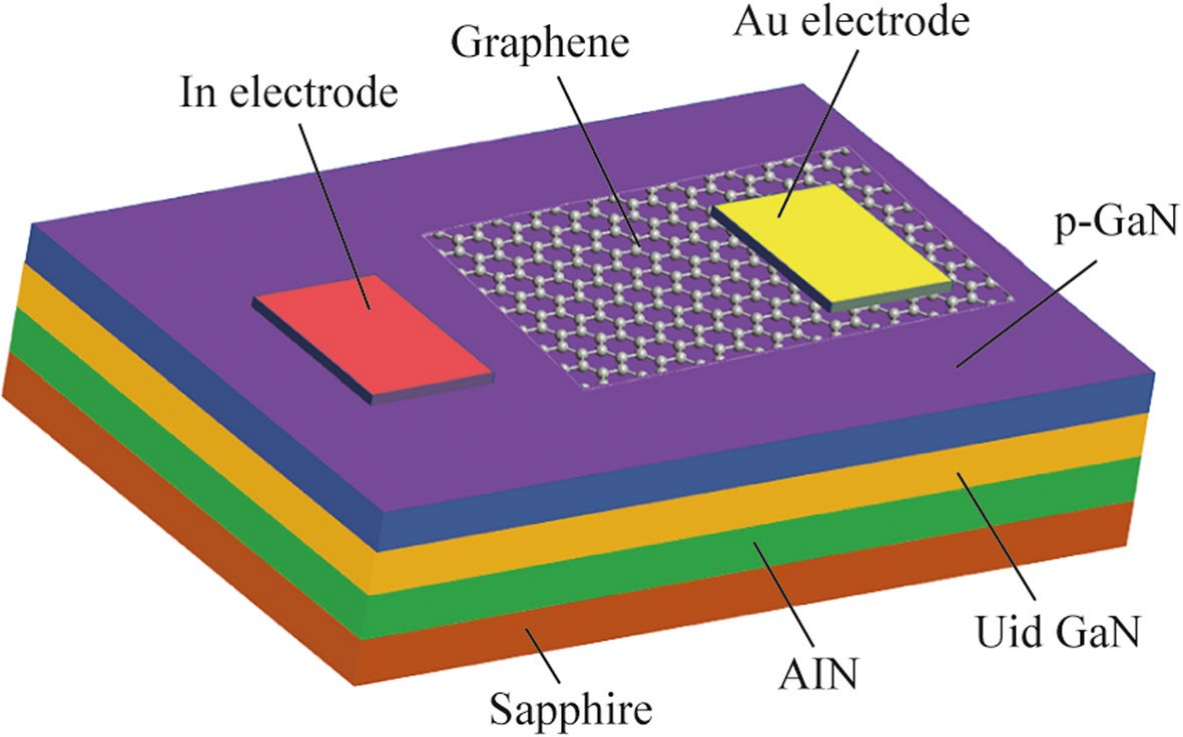
Schematic diagram of the fabricated PD based on ML graphene/GaN heterostructure

The device structure of the ML graphene/p-GaN PD is shown in Fig. 1. The structure of the GaN epitaxial film was grown by metal-organic chemical vapor deposition (MOCVD) on a sapphire substrate, which was composed of a 20 nm AlN buffer layer, a 1.8 μm unintentionally doped (UID) GaN epilayer, and a 300 nm Mg-doped p-type GaN. Before the transfer of graphene, the surface of GaN film was cleaned by acetone, isopropyl alcohol, H_2_SO_4_:HCl (1:1) solutions and deionized water [25–28]. The ML graphene was grown on sapphire by chemical vapor deposition (CVD). Then, the ML graphene was transferred to the GaN film by polymethylmethacrylate (PMMA) sacrificial layer, forming a heterostructure constructed by ML graphene and p-GaN. The ohmic contacts of p-GaN and graphene were obtained by evaporating In (100 nm) and Au (50 nm) electrodes with the electron beam, respectively. Ultimately, the PD device based on ML graphene/p-GaN heterostructure was successfully fabricated. During the graphene transfer and device fabrication, chemical pollution is inevitable. To avoid the white spots from residual chemical pollution, the fabricated PDs were conducted to be soaked in a miscible mixture of acetone and alcohol long enough.

The surface morphology and optical properties of the ML graphene and GaN substrate were characterized by atomic force microscope (AFM), Raman and photoluminescence (PL) spectra (LabRAM Inviamicro-Raman system). The electronic and optoelectronic properties of the PD based on graphene/GaN heterostructure were measured by a probe station equipped with a semiconductor parameter analyzer (Keithley 4200-SCS) under dark and illumination conditions.

Finally, the Quantum ATK software package based on the DFT was utilized to model and optimize the ML graphene/p-GaN heterostructure [29]. The method of PseudoDojo was adopted to optimize the structure, and the cut-off energy was 100 Hartree. The maximum interatomic stress was set to 0.01 eV/Å, and the maximum energy difference convergence limit was set to 10^−5^ eV during the optimization. To consider the influence of the van der Waals force in the heterostructure, DFT-D3 dispersion correction was added to the optimization calculation. Besides, a 20 Å vacuum layer was added in the *z* direction to avoid the influence of periodic interaction in the current transport direction.

## Results and discussion

**Fig. 2:**
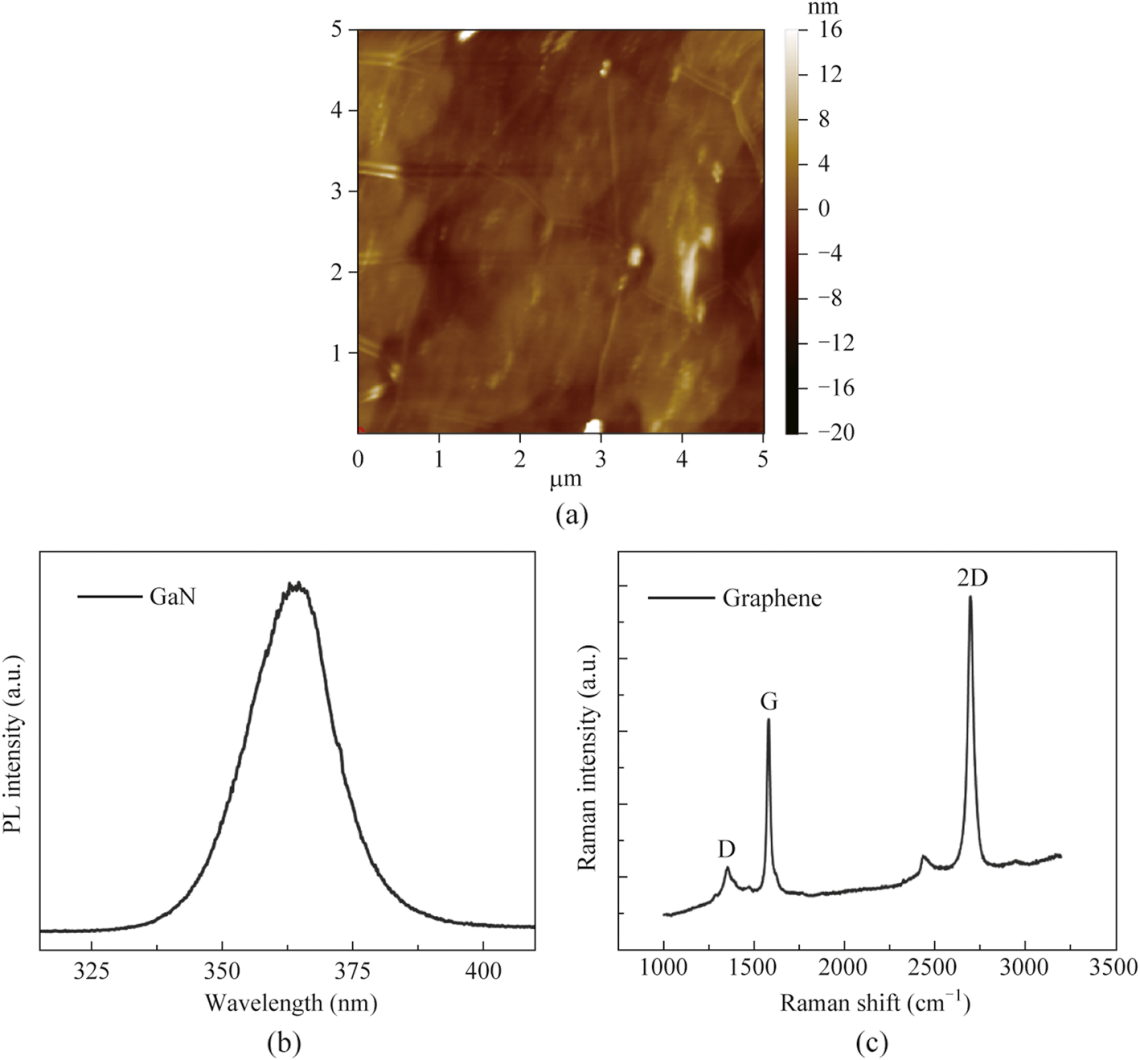
**a** Typical 5 μm × 5 μm AFM image of graphene transferred to GaN substrate. **b** PL spectra of p-GaN. **c** Raman spectra of the graphene film on GaN substrate

As shown in Fig. 2a, the AFM image of the ML graphene transferred to the GaN substrate shows some white spots and wrinkles, but there are no obvious holes or other defects in the graphene film. The white spots may come from residual chemical pollutants such as PMMA, and the wrinkles would be caused by atomic defects in graphene [30]. The surface root mean square (RMS) roughness of the ML graphene was obtained as 1.89 nm. Figure 2b shows the PL spectrum of GaN epitaxial film. It can be seen that there is a strong near-band-edge emission in the GaN PL spectrum, indicating the high crystalline quality of the GaN film. The PL peak of GaN is located near 365 nm, which is consistent with the band gap width of GaN. Figure 2c presents the Raman spectra of the graphene transferred to GaN film. The typical 2D band peak and G band peak are observed at wavenumbers of 2687 and 1582 cm^−1^, respectively, which are related to the transverse optical (TO) and longitudinal optical (LO) modes of 2D honeycomb lattice vibration generated by Γ point in Brillouin zone [25, 31]. The intensity ratio of the G peak to 2D peak is about 1:2, indicating the ML characteristic of the transferred graphene film [32, 33]. The D band peak near 1361 cm^−1^ can also be observed, indicating that the graphene has elastic electron–phonon scattering caused by wrinkles and disorders, which is consistent with the phenomenon observed in Fig. 2a. The adjoint Raman peak accompanying the 2D peak, according to our best knowledge, could be related to the impurity which was brought in when the graphene was transferred to the GaN.

**Fig. 3:**
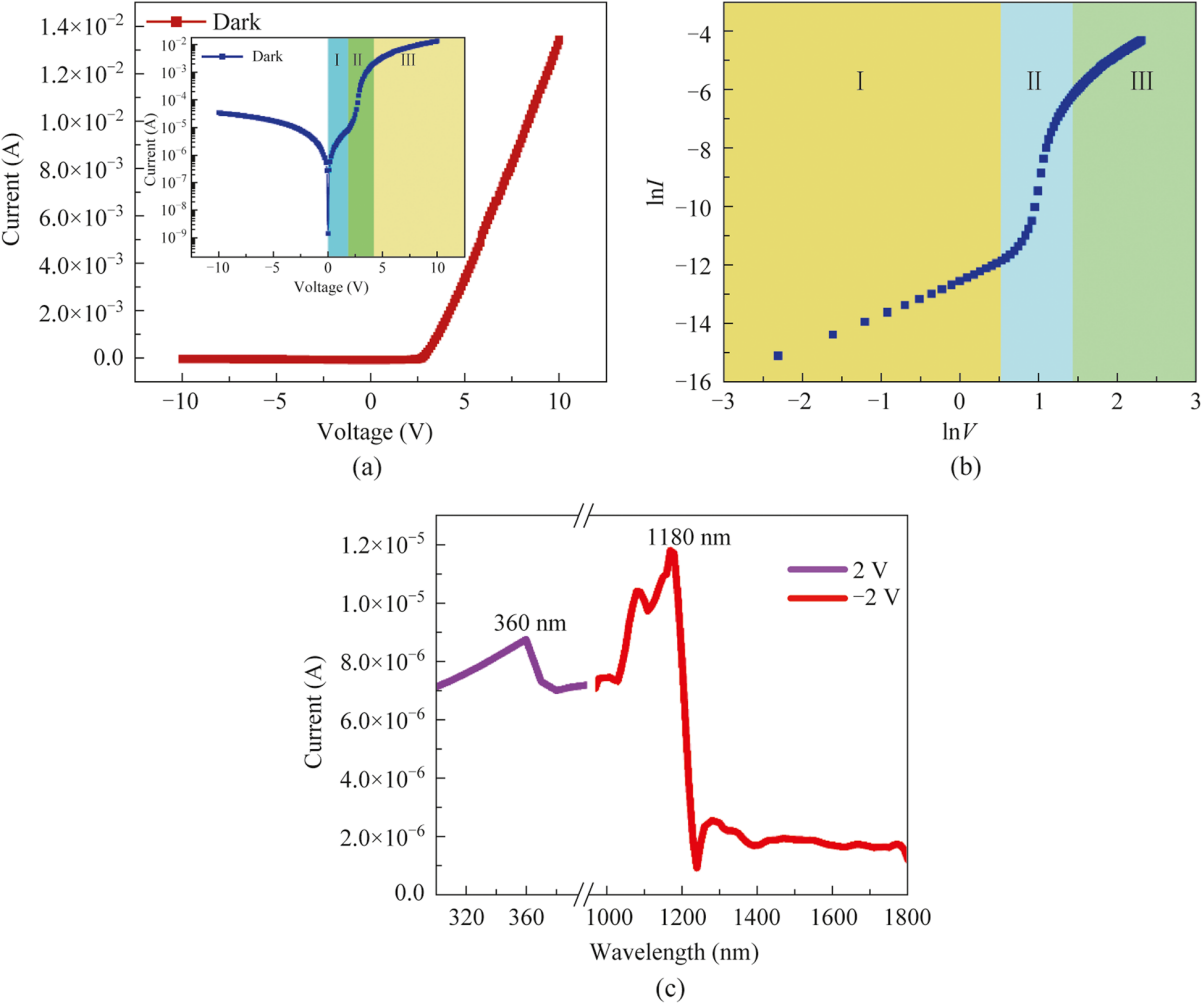
**a** *I–V* characteristic of the graphene/GaN heterostructure PD under dark condition. The inset shows the semi-logarithmic plot of *I–V* curve. **b** Double logarithmic plot of the *I–V* curve divulges three distinct regions of different trends. **c** Spectral response of the graphene/GaN PD

Figure 3a shows the* I–V* characteristics of the PD device based on ML graphene/p-GaN heterostructure under dark condition, which exhibits diode-like rectification behavior. Under forward bias up to 4 V, the current increases exponentially. As the bias was gradually increased, the current no longer exhibited the exponential characteristic, but instead increased linearly. To have a clear assessment of the *I–V* characteristics, a semi-logarithmic *I–V* diagram is plotted in the inset of Fig. 3a, which reveals three different regions of I, II, and III. As the forward bias continues to increase, there are different trends in the* I–V* diagram, which indicates the recombination mechanisms of the diode current. Moreover, Fig. 3b shows the double logarithmic diagram of the *I–V* curve to facilitate the study of the carrier transport mechanism under different biases. In region I, the main carriers are thermally generated electrons. In regional II, the injected carriers play a dominant role at a comparatively high bias voltage, resulting in space charge limited current (SCLC) transport. Furthermore, in region III, a slight slowdown in the upward trend indicates the existence of high-density trap states within the band gap [34]. To evaluate the performances of the PD device based on ML graphene/p-GaN, the photoresponse characteristics were measured. Figure 3c displays the spectral response of the PD device ranging in UV and IR wavelengths. As shown in Fig. 3c, the spectral response of 330−380 nm was observed at bias voltage of −2 V, and the device shows a sharp spectral cutoff of ~360 nm, which is consistent with the PL emission peak of GaN shown in Fig. 2b. In addition, the infrared response in the range of 1000−1210 nm was also observed at 2 V, which reveals a sharp cutoff of ~1180 nm. The spectral responses convincingly demonstrate that the realization of UV-IR dual-wavelength PD device based on ML graphene/p-GaN heterostructure.

**Fig. 4:**
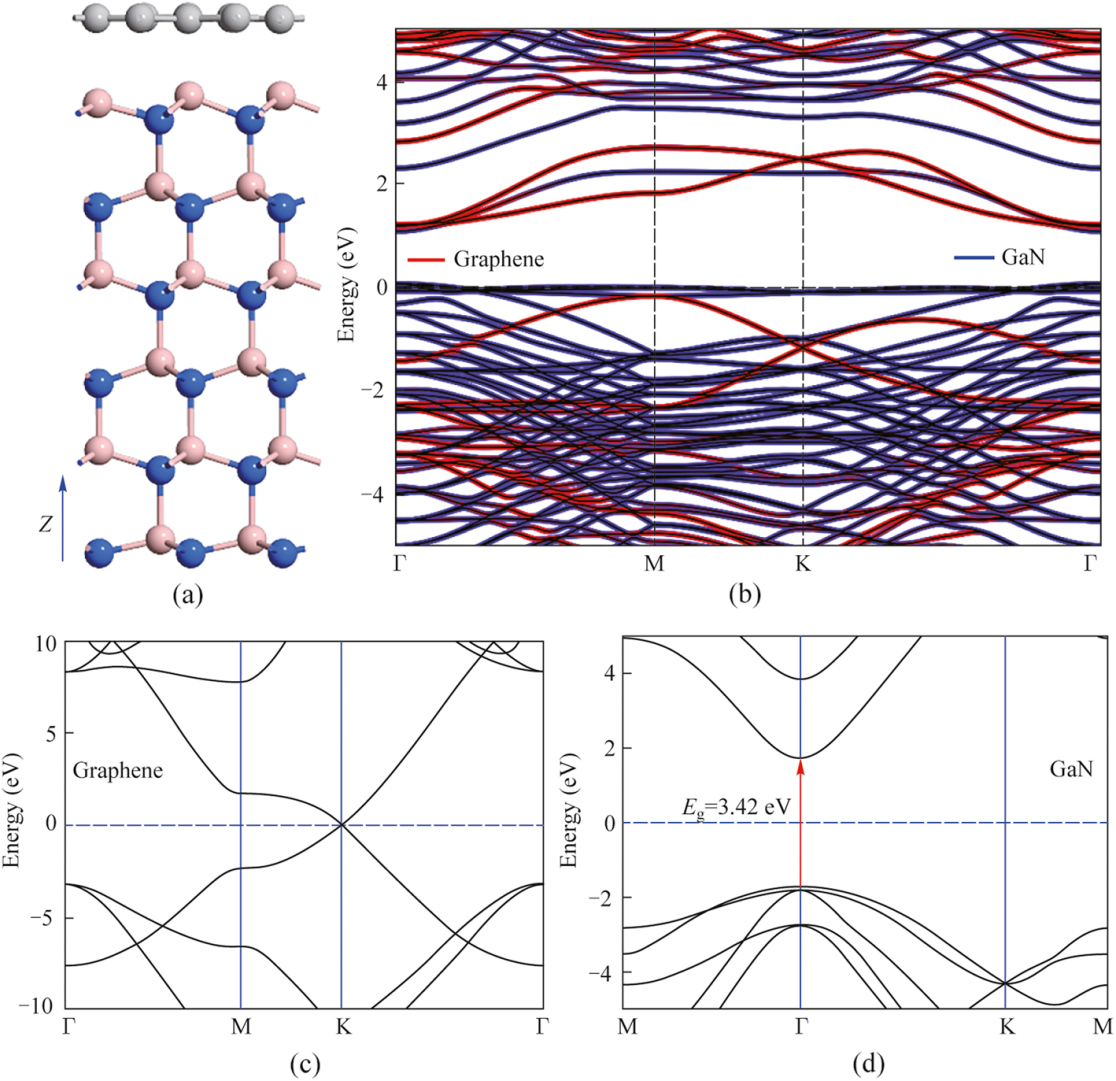
**a** Side view of the ML graphene/GaN vertical stacked heterostructure. **b** Projected energy band structure of the graphene/GaN heterostructure. The calculated electric band structure of the pristine **c** ML graphene and **d** bulk GaN

Furthermore, the ML graphene/p-GaN heterostructure was constructed by DFT calculations to theoretically investigate its electronic and optical properties. Figure 4a shows a side view of the optimized ML graphene/p-GaN hybrid structure with the corresponding vertical interlayer distance of 2.4 Å in the out-of-plane direction. The energy band structure of the 2 × 2 graphene/$\sqrt{3}$×$\sqrt{3}$ (0001) GaN is presented in Fig. 4b. Figure 4c and d show the energy band structures of the pristine ML graphene and bulk GaN, respectively. Both the conduction band minimum (CBM) and valence band maximum (VBM) of the ML graphene almost intersect at the Fermi level (Dirac cone). The direct bandgap of bulk GaN is 3.42 eV, and VBM and CBM appear at the Γ-point of Brillion zone. The above results are reliable and consistent with the experimental reports [35]. As the GGA-PBE usually underestimates the bandgap, the GGA-1/2 method has been employed for a more accurate bandgap, which corrects the DFT self-interaction error by defining an atomic self-energy potential that cancels the electron–hole self-interaction energy [36].

**Fig. 5:**
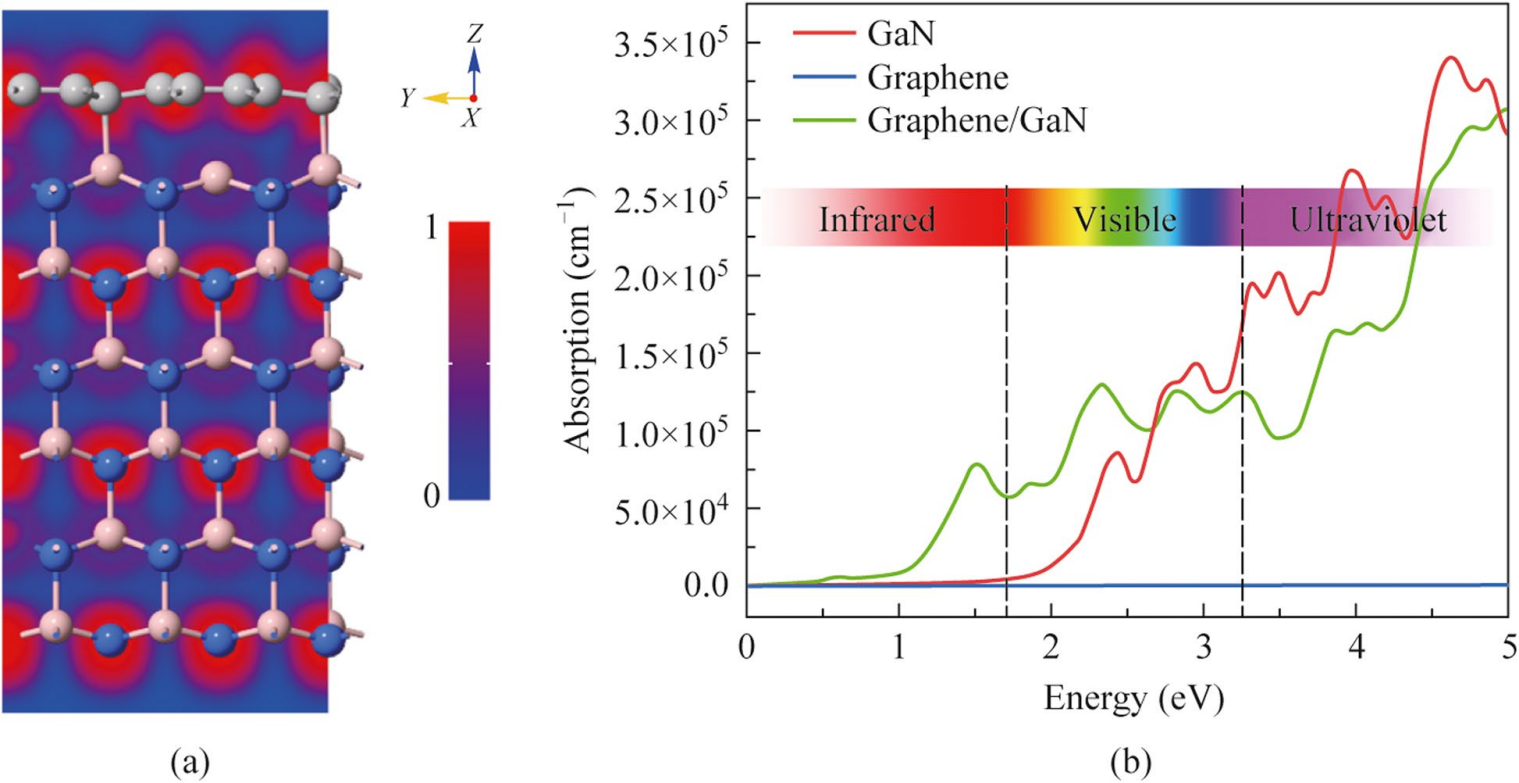
**a** ELF plot of the graphene/GaN hybrid heterostructure. **b** Optical absorption coefficient of pristine ML graphene, bulk GaN, and graphene/GaN heterostructure as a function of photo energy

Additionally, in order to study the binding characteristic between 2D graphene and bulk GaN, we have plotted the electronic local function (ELF) of the ML graphene/p-GaN heterostructure, as shown in Fig. 5a. It illustrates that the ML graphene transferred on p-GaN substrate exhibits strong electron localization. Figure 5b shows the optical absorption coefficients of the pristine ML graphene, bulk GaN, and hybrid graphene/GaN heterostructure as a function of photon energy. It can help to better understand the difference of optical properties between hybrid heterostructures and pristine materials. It can be clearly observed that the optical absorption of graphene/GaN hybrid heterojunction in the IR region is significantly higher than that of the pristine material. This result shows that graphene/GaN heterojunction can have both UV and IR responses, which provides a potential strategy for the realization of high-performance dual-wavelength PD. Here, it should be noted that, as shown in Fig. 5b, comparing to ML graphene and bulk GaN, the behavior of optical absorption of graphene/GaN heterostructure is quite different. Within short wavelength, both the graphene and GaN can be excited by photons. Due to the ultra-thin thickness of the graphene, the UV photoresponse of the heterojunction is responsible for the GaN layer. Meanwhile, at long wavelength, the GaN is absent from photon excitation and, then, the absorption from the graphene is dramatically enhanced for the coupling effects from the graphene/GaN heterostructure, which is in stark contrast with the absorption of the ML graphene.

**Fig. 6:**
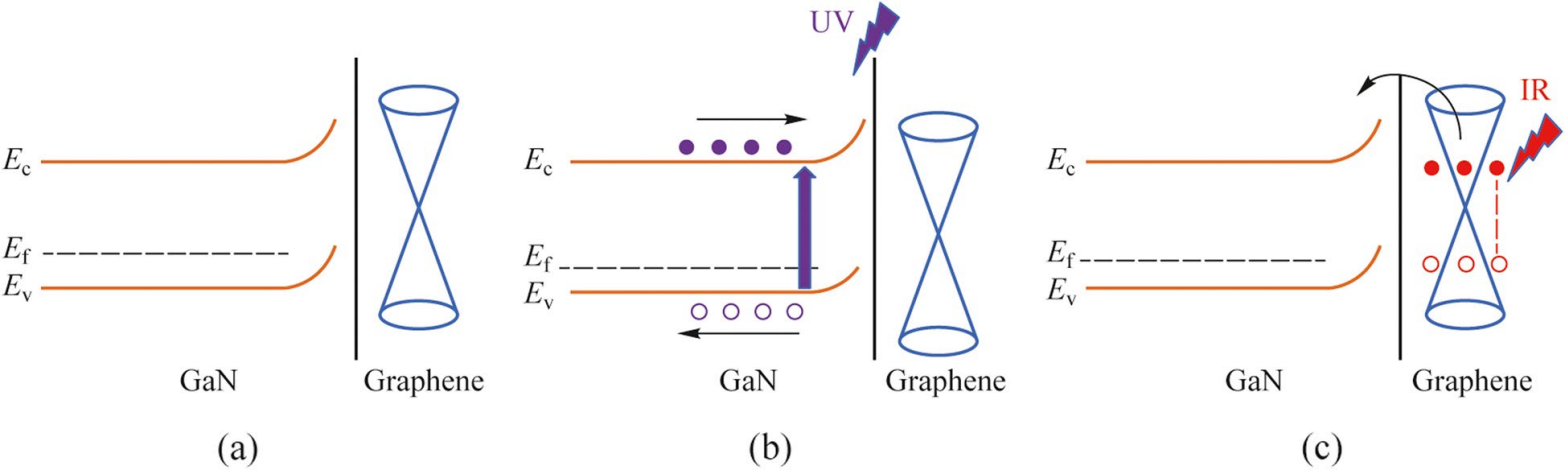
Energy band diagram of the ML graphene/p-GaN heterostructure under **a** dark, **b** UV light, and **c** IR light illumination

Finally, the energy band diagram of the ML graphene/p-GaN is depicted in Fig. 6. Figure 6a shows the energy band without light illumination, the potential barrier between graphene and GaN decreases with increasing forward bias, as shown in Fig. 3a where the current increases exponentially. While at the reverse bias, the height of the barrier increases and the depletion region widens. As shown in Fig. 6b, when the incident UV light is illuminated on the heterostructure, the UV photon energy is greater than the band gap of GaN, resulting in electron–hole pairs that are rapidly separated by the built-in electric field at the graphene/p-GaN interface. Electrons leap over the potential barrier into the graphene and are collected by the electrodes to produce the output electrical signals. As for graphene, the Fermi level density of states is relatively low, which will be significantly affected by bias [37]. At positive bias, the Fermi level of graphene moves upwards. As shown in Fig. 6c, with the decrease of the barrier height at forward bias, graphene produces photogenerated carriers under IR light illumination and leaps over the barrier to form a photocurrent. However, the IR photon energy is less than the band gap of GaN, so it is impossible to generate photoresponses in the UV wavelengths. The explanations agree well with the above experimental results of UV-IR dual-wavelength photoresponse for the ML graphene/p-GaN displayed in Fig. 3c.

## Conclusion

In summary, the UV-IR dual-wavelength PD based on ML graphene/p-GaN hybrid heterostructure has been successfully prepared. The AFM, PL, and Raman characterizations confirmed the morphological and optical properties of the graphene/GaN heterostructure. Furthermore, the fabricated PD device exhibited spectral responses in the two wavelength ranges of 330−380 and 1000−1210 nm, with the sharp spectral cutoffs located at ~360 and ~1180 nm, respectively. In addition, the dual-wavelength photoresponses characteristic of graphene/GaN heterostructure was explained by theoretical DFT calculations. Consequently, the ML graphene/GaN heterostructure is expected to be a promising candidate for the development of UV-IR dual-wavelength optoelectronic devices.

## Data Availability

The data that support the findings of this study are available from the corresponding author upon reasonable request.

## References

[1] Zeng C, Lin W, He T, Zhao Y, Sun Y, Cui Q, Zhang X, Lu S, Zhang X, Xu Y, Kong M, Zhang B (2020). Ultraviolet-infrared dual-color photodetector based on vertical GaN nanowire array and graphene. Chin. Opt. Lett..

[2] Perera AGU, Ariyawansa G, Rinzan MBM, Stevens M, Alevli M, Dietz N, Matsik SG, Asghar A, Ferguson IT, Luo H, Bezinger A, Liu HC (2007). Performance improvements of ultraviolet/infrared dual-band detectors. Infrared Phys. Technol..

[3] Guo J, Ye B, Gu Y, Liu Y, Yang X, Xie F, Zhang X, Qian W, Zhang X, Lu N, Yang G (2023). Broadband photodetector for ultraviolet to visible wavelengths based on the Ba_2_PbI_4_/GaN heterostructure. ACS Appl. Mater. Interfaces.

[4] Liu H, Ye B, Gu Y, Liu Y, Yang X, Xie F, Zhang X, Qian W, Zhang X, Lu N, Yang G (2023). UV-visible dual-band photodetector based on an all-inorganic Mn-doped CsPbCl_3_/GaN type-II heterojunction. Appl. Phys. Lett..

[5] Ye BJ, Liu YS, Xie F, Yang XF, Gu Y, Zhang XM, Qian WY, Zhu C, Lu NY, Chen GQ, Yang GF (2023). Dual-wavelength photodetector based on layered WSe_2_/n-GaN van der Waals heterostructure. Mater. Today Nano.

[6] Qi L, Li X, Tang Z, Yin S, Zhao Y (2014). Monolithically integrated UV/IR dual-color photodetector with AlGaN/GaN heterojunction structure. Semicond. Technol..

[7] Singh DK, Pant RK, Nanda KK, Krupanidhi SB (2021). Differentiation of ultraviolet/visible photons from near infrared photons by MoS_2_/GaN/Si-based photodetector. Appl. Phys. Lett..

[8] Sandhu HK, John JW, Jakhar A, Sharma A, Jain A, Das S (2022). MoSe2/n-GaN heterojunction induced high photoconductive gain for low-noise broadband photodetection from ultraviolet to near-infrared wavelengths. Adv. Mater. Interfaces..

[9] Solanke SV, Soman R, Rangarajan M, Raghavan S, Nath DN (2021). UV/near-IR dual band photodetector based on p-GaN/α-In_2_Se_3_ heterojunction. Sens. Actuator A Phys..

[10] Tang X, Hao Z, Wang L, Yu J, Wang X, Luo Y, Sun C, Han Y, Xiong B, Wang J, Li H (2022). Plasmon-enhanced hot-electron photodetector based on Au/GaN-nanopillar arrays for short-wave-infrared detection. Appl. Sci. (Basel).

[11] Zhang X, Liu B, Liu Q, Yang W, Xiong C, Li J, Jiang X (2017). Ultrasensitive and highly selective photodetections of UV-A rays based on individual bicrystalline GaN nanowire. ACS Appl. Mater. Interfaces.

[12] Rabiee Golgir H, Li DW, Keramatnejad K, Zou QM, Xiao J, Wang F, Jiang L, Silvain JF, Lu YF (2017). Fast growth of GaN epilayers via laser-assisted metal-organic chemical vapor deposition for ultraviolet photodetector applications. ACS Appl. Mater. Interfaces.

[13] Guo J, Gu Y, Liu Y, Liang F, Chen W, Xie F, Yang X, Qian W, Zhang X, Chen G, Yang G (2023). Polarization assisted interdigital AlGaN/GaN heterostructure ultraviolet photodetectors. IEEE Trans. Electron Dev..

[14] Gong B, Ye B, Gu Y, Xie F, Zhang X, Qian W, Zhang X, Lu N, Yang G (2024). Self-powered GaN-based MSM ultraviolet photodetector with asymmetrical interdigitated structure. IEEE Trans. Electron Dev..

[15] Tian H, Liu Q, Hu A, He X, Hu Z, Guo X (2018). Hybrid graphene/GaN ultraviolet photo-transistors with high responsivity and speed. Opt. Express.

[16] Gundimeda A, Krishna S, Aggarwal N, Sharma A, Sharma ND, Maurya KK, Husale S, Gupta G (2017). Fabrication of non-polar GaN based highly responsive and fast UV photodetector. Appl. Phys. Lett..

[17] Yang J, Tang L, Luo W, Shen J, Zhou D, Feng S, Wei X, Shi H (2019). Light trapping in conformal graphene/silicon nanoholes for high-performance photodetectors. ACS Appl. Mater. Interfaces.

[18] Xie C, Wang Y, Zhang ZX, Wang D, Luo LB (2018). Graphene/semiconductor hybrid heterostructures for optoelectronic device applications. Nano Today.

[19] Wei X, Yan FG, Lv QS, Shen C, Wang KY (2017). Fast gate-tunable photodetection in the graphene sandwiched WSe_2_/GaSe heterojunctions. Nanoscale.

[20] Deb P, Dhar JC (2019). Fast response UV photodetection using TiO_2_ nanowire/graphene oxide thin-film heterostructure. IEEE Photonics Technol. Lett..

[21] Nowak D, Clapa M, Kula P, Sochacki M, Stonio B, Galazka M, Pelka M, Kuten D, Niedzielski P (2019). Influence of the interactions at the graphene-substrate boundary on graphene sensitivity to UV irradiation. Materials (Basel)..

[22] Guo X, Wang W, Nan H, Yu Y, Jiang J, Zhao W, Li J, Zafar Z, Xiang N, Ni Z, Hu W, You Y, Ni Z (2016). High-performance graphene photodetector using interfacial gating. Optica.

[23] Hu W, Ye Z, Liao L, Chen H, Chen L, Ding R, He L, Chen X, Lu W (2014). 128 × 128 long-wavelength/mid-wavelength two-color HgCdTe infrared focal plane array detector with ultralow spectral cross talk. Opt. Lett..

[24] He T, Ma H, Wang Z, Li Q, Liu S, Duan S, Xu T, Wang J, Wu H, Zhong F, Ye Y, Wu J, Lin S, Zhang K, Martyniuk P, Rogalski A, Wang P, Li L, Lin H, Hu W (2024). On-chip optoelectronic logic gates operating in the telecom band. Nat. Photonics..

[25] Kim S, Seo TH, Kim MJ, Song KM, Suh EK, Kim H (2015). Graphene-GaN Schottky diodes. Nano Res..

[26] Seo TH, Lee KJ, Oh TS, Lee YS, Jeong H, Park AH, Kim H, Choi YR, Suh EK, Cuong TV, Pham VH, Chung JS, Kim EJ (2011). Graphene network on indium tin oxide nanodot nodes for transparent and current spreading electrode in InGaN/GaN light emitting diode. Appl. Phys. Lett..

[27] Hoon Seo T, Kyoung Kim B, Shin G, Lee C, Jong Kim M, Kim H, Suh EK (2013). Graphene-silver nanowire hybrid structure as a transparent and current spreading electrode in ultraviolet light emitting diodes. Appl. Phys. Lett..

[28] Cho H, Lee C, Oh SI, Park S, Kim HC, Kim MJ (2012). J, K.M.: Parametric study of methanol chemical vapor deposition growth for graphene. Carbon. Lett..

[29] Smidstrup S, Markussen T, Vancraeyveld P, Wellendorff J, Schneider J, Gunst T, Verstichel B, Stradi D, Khomyakov PA, Vej-Hansen UG, Lee ME, Chill ST, Rasmussen F, Penazzi G, Corsetti F, Ojanperä A, Jensen K, Palsgaard MLN, Martinez U, Blom A, Brandbyge M, Stokbro K (2020). Quantum ATK: an integrated platform of electronic and atomic-scale modelling tools. J. Phys. Condens. Matter..

[30] Han GH, Güneş F, Bae JJ, Kim ES, Chae SJ, Shin HJ, Choi JY, Pribat D, Lee YH (2011). Influence of copper morphology in forming nucleation seeds for graphene growth. Nano Lett..

[31] Ferrari AC, Meyer JC, Scardaci V, Casiraghi C, Lazzeri M, Mauri F, Piscanec S, Jiang D, Novoselov KS, Roth S, Geim AK (2006). Raman spectrum of graphene and graphene layers. Phys. Rev. Lett..

[32] Yang F, Cong H, Yu K, Zhou L, Wang N, Liu Z, Li C, Wang Q, Cheng B (2017). Ultrathin broadband germanium-graphene hybrid photodetector with high performance. ACS Appl. Mater. Interfaces.

[33] Wei X, Yan F, Lv Q, Zhu W, Hu C, Patanè A, Wang K (2019). Enhanced photoresponse in MoTe_2_ photodetectors with asymmetric graphene contacts. Adv. Opt. Mater..

[34] Yüksel ÖF, Kuş M, Şimşir N, Şafak H, Şahin M, Yenel E (2011). A detailed analysis of current-voltage characteristics of Au/perylene-monoimide/n-Si Schottky barrier diodes over a wide temperature range. J. Appl. Phys..

[35] Liu C, Li E, Zheng Y, Bai K, Cui Z, Ma D (2021). Regulation of vertical and biaxial strain on electronic and optical properties of G-GaN-G sandwich heterostructure. J. Mater. Sci..

[36] Ferreira LG, Marques M, Teles LK (2008). Approximation to density functional theory for the calculation of band gaps of semiconductors. Phys Rev B Condens Matter Mater Phys..

[37] Wu Z, Lu Y, Xu W, Zhang Y, Li J, Lin S (2016). Surface plasmon enhanced graphene/p-GaN heterostructure light-emitting-diode by Ag nano-particles. Nano Energy.

